# Geriatric rehabilitation care after hip fracture

**DOI:** 10.1007/s41999-023-00755-4

**Published:** 2023-02-14

**Authors:** G. F. Mattiazzo, Y. M. Drewes, M. van Eijk, W. P. Achterberg

**Affiliations:** 1 Department of Public Health and Primary Care, Leiden University Medical Center, Leiden, The Netherlands; 2 Department of Internal Medicine, Section Gerontology and Geriatrics, Leiden University Medical Center, Leiden, The Netherlands; 3 University Network for the Care Sector Zuid-Holland, Leiden University Medical Center, Leiden, The Netherlands; 4 Department Internal Medicine Section Geriatrics, Haaglanden Medisch Centrum, The Hague, the Netherlands

**Keywords:** Geriatric rehabilitation, Standard care, Hip fractures, Care pathways

## Abstract

**Purpose:**

After acute hospital admission, patients with a hip fracture are frequently discharged to skilled nursing homes providing geriatric rehabilitation (GR). There are few evidence-based studies regarding specific treatment times and assessments during GR. This study aims to provide a description of care for hip fracture patients during GR in the Netherlands.

**Methods:**

Descriptive study analyzing the care pathways from GR facilities, regarding healthcare professionals involved, allocated treatment time per profession, total length of rehabilitation stay, and assessment instruments. Based on the reimbursement algorithm (diagnostic treatment combination = DBCs), of 25 patients, the registered actual treatment time per profession was calculated.

**Results:**

The care pathways pivoted on three groups of health care professionals: medical team (MT), physiotherapy (PT), and occupational therapy (OT). There was some discrepancy between the allocated time in the care pathways and the calculated mean actual treatment time from the DBCs. First week: MT 120–180 min, DBC 120 (SD: 59) minutes; PT 120–230 min, DBC 129 (SD: 58) minutes; and OT 65–165 min, DBC 93 (SD: 61) minutes. From week two onwards, MT 15–36 min, DBC 49 (SD: 29) minutes; PT 74–179 min, DBC 125 (SD: 50) minutes; and OT 25–60 min, DBC 47 (SD: 44) minutes. Dieticians, psychologists, and social workers were sporadically mentioned. There was heterogeneity in the assessment and screening tools.

**Conclusions:**

It is difficult to define current standard care in GR after hip fracture in the Netherlands due to the diversity in care pathways and large practice variation. This is a problem in conducting randomized effectiveness research with care provided as control.

**Trial register and date of registration:**

NL7491 04-02-2019.

## Key summary points

**Aim:**

To describe the care provided in the Netherlands in geriatric rehabilitation (GR) after a hip fracture, using care pathways and diagnosis treatment combinations from various geriatric rehabilitation facilities.

**Findings:**

Care provided in GR after hip fracture is difficult to define due to the diversity in care pathways and large practice variation.

**Message:**

Further research is needed to investigate whether a standardized care pathway is effective for GR.

## Introduction

The prognosis of patients with hip fractures is a major concern because the 1-year mortality rate is 20% [1, 2]. In addition, 60–80% of these patients do not reach their pre-morbid independence, and approximately, 20% of them are permanently institutionalized [3]. This leads to great personal, financial, and societal burden [4]. Optimizing functional recovery after a hip fracture is, therefore, of great importance in the older population. In the Netherlands, approximately 17,000 older patients with hip fractures are registered yearly, and the mortality rate is comparable to the rates mentioned in international literature [5–7]. Most international research has focused on the acute phase after a hip fracture. This has resulted in a relatively standardized surgical procedure followed by perioperative care during the in-hospital stay of several days to weeks [8]. Furthermore, it has led to incorporating inpatient comprehensive geriatric assessment and the introduction of orthogeriatric care units, which has proved to decrease mortality [9, 10]. These evidence-based practices have been incorporated into guidelines for standardized care and subsequently translated into clinical care pathways [11]. They provide guidance regarding the care during the acute phase after a hip fracture.

After hospitalization, averaging 8.6 days in the Netherlands, most patients are discharged for further rehabilitation [12–14]. Rehabilitation after a hip fracture is initiated in the clinical phase after surgery and continues in skilled geriatric rehabilitation (GR) for patients who score high on frailty and complex multimorbidity, or in the home setting when they are not frail. However, in literature, care pathways for hip fracture patients in the post-acute phase focus almost solely on the immediate discharge destination after an acute care stay and less on the actual geriatric rehabilitation process [15, 16]. Evidence for GR after a hip fracture is available, but relatively scarce due to the limited number of empirical, high quality effect studies regarding specific treatments, procedures, practices, services, and approaches [4, 17]. In addition, there is a large variation in assessment instruments used in GR to define and monitor functional recovery, resulting in a wide variety of outcomes in research ranging from psychosocial aspects to biological outcomes [2, 18]. Due to this variety, we have little insight into GR care after hip fractures [18]. This makes comparisons with other studies, countries, or settings difficult. Care pathways may represent usual care, but they tend to vary in key components among various countries, and it is not known whether they also represent actual care [19].

In the Netherlands, after acute hospital admission, 55% of the patients with a hip fracture are discharged to GR facilities in nursing homes, in which elderly care physician specifically trained in rehabilitation lead the team [15, 20]. The aim of this study is to provide a description of the care provided in the GR facilities in the Netherlands. This will be based on the therapy time described in the care pathway and the actual contribution of different healthcare professionals in the rehabilitation using the mandated time registration for reimbursement purposes, the Diagnosis Treatment Combinations (DBC) [21]. This provides information about the variety between facilities, and how care pathway schemes are executed in everyday practice.

## Methods

### Study design and setting

The data we collected were derived from the Inventarisation of Prognostic factors and their Contribution towArds REhabilitation in older persons study (HIPCARE). HIPCARE is an inception cohort-based study, initiated in 2018 and still ongoing, of patients with hip fractures admitted to the acute care at Haaglanden Medical Centre (HMC +) in the Hague [3]. We included previously community-dwelling patients aged 70 years or older with a unilateral hip fracture and who were eligible for (inpatient) geriatric rehabilitation. The exclusion criteria were patients previously residing in nursing homes, being younger than 70 years of age, having a pathological hip fracture, being unwilling or unable to provide informed consent, having insufficient mastery of the Dutch language, and patients already included in this study due to a prior hip fracture. After an acute hospital setting, patients were either discharged to a geriatric rehabilitation setting or to their own home. For this descriptive study, we selected only patients that were discharged to geriatric rehabilitation facilities.

### Data collection

During the period March 2020 to June 2021, retrospective data were requested from eight GR facilities that received patients (post-acute hip fracture patients) of the HIPCARE study admitted into GR from December 2018 to November 2020. All patients, at the time of data collection, had finished the GR program and were discharged home. We contacted the research coordinator, manager, or physiotherapist working in the GR to collect hip fracture care pathways and DBCs from the GR facilities. For the DBC registration for reimbursement, we focused on the first five HIPCARE patients in each GR facility who completed the GR and were subsequently discharged home. In the Netherlands, DBC is a compulsory registration for insurance purposes regarding the time that is used by healthcare professionals involved. It provides information about healthcare professionals consulted and the treatment administered in minutes per healthcare professional per week per patient during GR [22]. Providing this information for research purposes is not compulsory.

### Analysis

Two researchers (GFM and YMD) reviewed the care pathways*.* Predefined items such as healthcare professionals consulted, allocated length of rehabilitation trajectory, allocated treatment intensity in minutes during rehabilitation, and allocated frequency of multidisciplinary consultation meetings were registered. Furthermore, standardized assessment instruments for screening, diagnosis, and evaluation of progression that were described were extracted for comparison between the care pathways. The DBCs provided information on diagnosis, total treatment time in minutes, and length of stay in days. Total treatment time is defined as the combined treatment time reported by all healthcare professionals for each individual patient. From this information, the average treatment time actually given per week and day were calculated per participant.

In addition, the weekly treatment time provided by each healthcare professional as recorded in the DBC registration was collected. The planned time as was described in the care pathway was then compared to the (mean, SD) actual treatment time given according to the DBC registration. The first week of GR is separately calculated and documented, because during this week, admission examinations are also included in the treatment time, next to regular treatment. This is associated with higher registered treatment times.

### Ethics

The HIPCARE study was approved by the medical ethics committee of Leiden/Den Haag/Delft (protocol number 18-081, NL66871.098.18) and published in the Netherlands Trial Registry (NTR) (trial registration number NL7491). All patients gave their written permission to obtain information about their rehabilitation care, including access to data from the DBCs.

## Results

### Data collection: GR care pathways and DBCs

Six of the eight GR facilities, A, B, C, D, E and F used standardized care pathways created by the GR facility itself. Facility A had a care pathway with a length of 5 weeks. Facility B had two care pathways, one for 6 weeks and one for 10 weeks. Facilities C and D had care pathways with a length of 6 weeks. Facility E had one care pathway with a length of 6 weeks. Facility F had three care pathways, one for 6 weeks, one for 10 weeks and one for 20 weeks. Facility G used individualized treatment plans based on the International Classification of Functioning, Disability and Health (ICF) coding system and facility H had no documented care pathways (Appendix A).The different care pathways of GR facilities B and F did not contain information about the patient groups with an intended longer stay.

Only 25 DBC registrations could be retrieved from seven of the eight GR facilities. Three of these GR facilities had received the most patients and were able to submit the DBC registrations of the first five patients. The other five GR facilities received only one to four patients in the relevant time frame. Table 1 shows the 25 DBC registrations and care pathways from eight GR facilities that were used for the analysis.

**Table 1:** Retrieved care pathways and DBC registration with number of participants per geriatric rehabilitation facility

GR facility (*n* = 8)	CPs			DBC registration	
	Amount of CPs	Amount of CPs with allocated treatment time in minutes	Amount of CPs with description of screening tools	Amount of patients with registered total treatment time	Amount of patients with registered treatment time per healthcare professional
A	1	1	1	4	4
B	2	2	0	5	5
C	1	1	0	0	0
D	1	1	1	1	1
E	1	1	1	2	2
F	3	0	1	5	0
G	0	0	0	5	5
H	0	0	0	3	0
Total	9	6	4	25	17

*GR* Geriatric rehabilitation, *CP* Care pathways, *DBC* Diagnose treatment combination (reimbursement system of the Netherlands)

### Therapy intensity from the care pathways

Six GR facilities indicated that, apart from the specialized nursing staff, GR pivots on three main healthcare professionals: the medical team, the occupational therapist, and the physiotherapist. Appendix A shows the therapy intensity as described per care pathway per healthcare professional per week. Four of the six GR facilities registered a combination of individual as well as group therapy. The remaining two GR facilities distinguished between individual and group therapy provided by the physiotherapist. Unfortunately, the frequency of group therapy was not specified. To avoid the possibility that the calculated individual and group therapy were outliers in these two GR facilities, only physiotherapy on an individual basis was considered in the analysis. Healthcare professionals such as psychologists, dieticians, speech therapists, and social workers were available on a consultation basis and were not standard in the care pathways. Five GR facilities described they had multidisciplinary consultation meetings to evaluate progression and adjust the rehabilitation where and if necessary. In addition to the intensity of therapy GR facility A, D, E and F described the content of therapy based on assessment and screening tools. The tools selected by the facilities varied. GR facility A, D, and F applied the Utrecht Scale for Evaluation of Clinical Rehabilitation (USER) for the evaluation of cognitive (dis)abilities, pain, mobility, and Activity of Daily Living (ADL), as well as the Canadian Occupational Performance Measure (COPM) for the evaluation of problems with ADL, participation, and goal setting. Next to the USER, the additional assessment tools for mobility in GR A and D were Time Up and Go (TUG), 10-m walk test (10MWT) and Functional Ambulation Categories (FAC). Risk inventory and evaluation was incorporated in GR facilities A, D and F, using the Visual Analogue Scale (VAS) for screening of pain and Short Nutritional Assessment Questionnaire (SNAQ) to screen for malnutrition. GR facility D additionally screened for polypharmacy and falls. GR facilities D and E centered on caregiver involvement by adapting the caregiver scan or caregiver strain index (CSI) (see Appendix B).

### Treatment description based on DBC registration of 25 patients

**Fig. 1:**
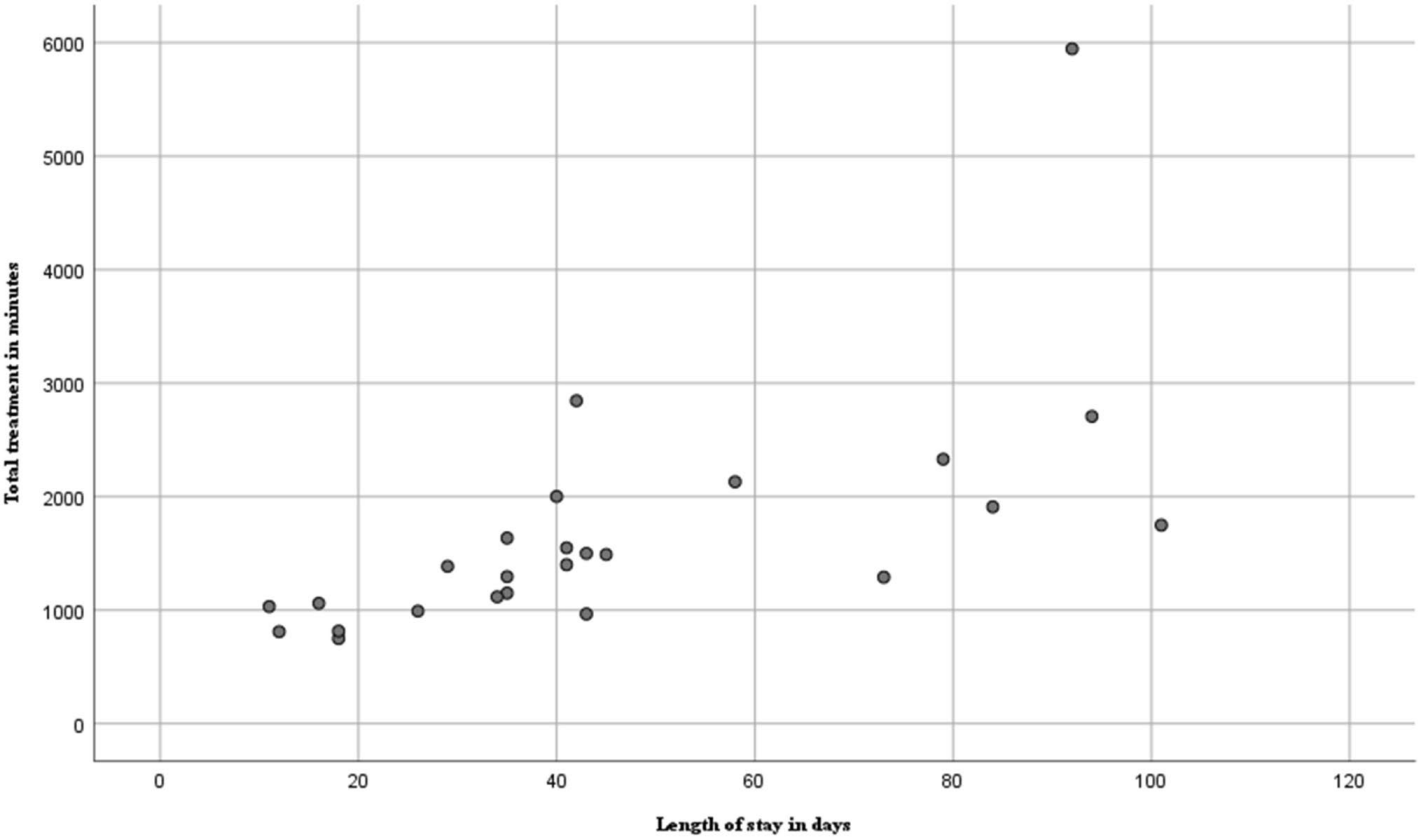
Total treatment time in minutes per participant related to length of stay in geriatric rehabilitation facilities (*n* = 25 patients). Total treatment time is defined as the total combined amount of treatment given by all healthcare professionals. Length of stay is indicated in the DBCs

Figure 1 illustrates the total treatment time in minutes compared to the total length of stay per patient. Sixteen of the 25 patients (64%) were discharged within 6 weeks of which 10 patients were discharged between 4 and 6 weeks. Nine patients (36%) were discharged after 6 weeks.

**Fig. 2:**
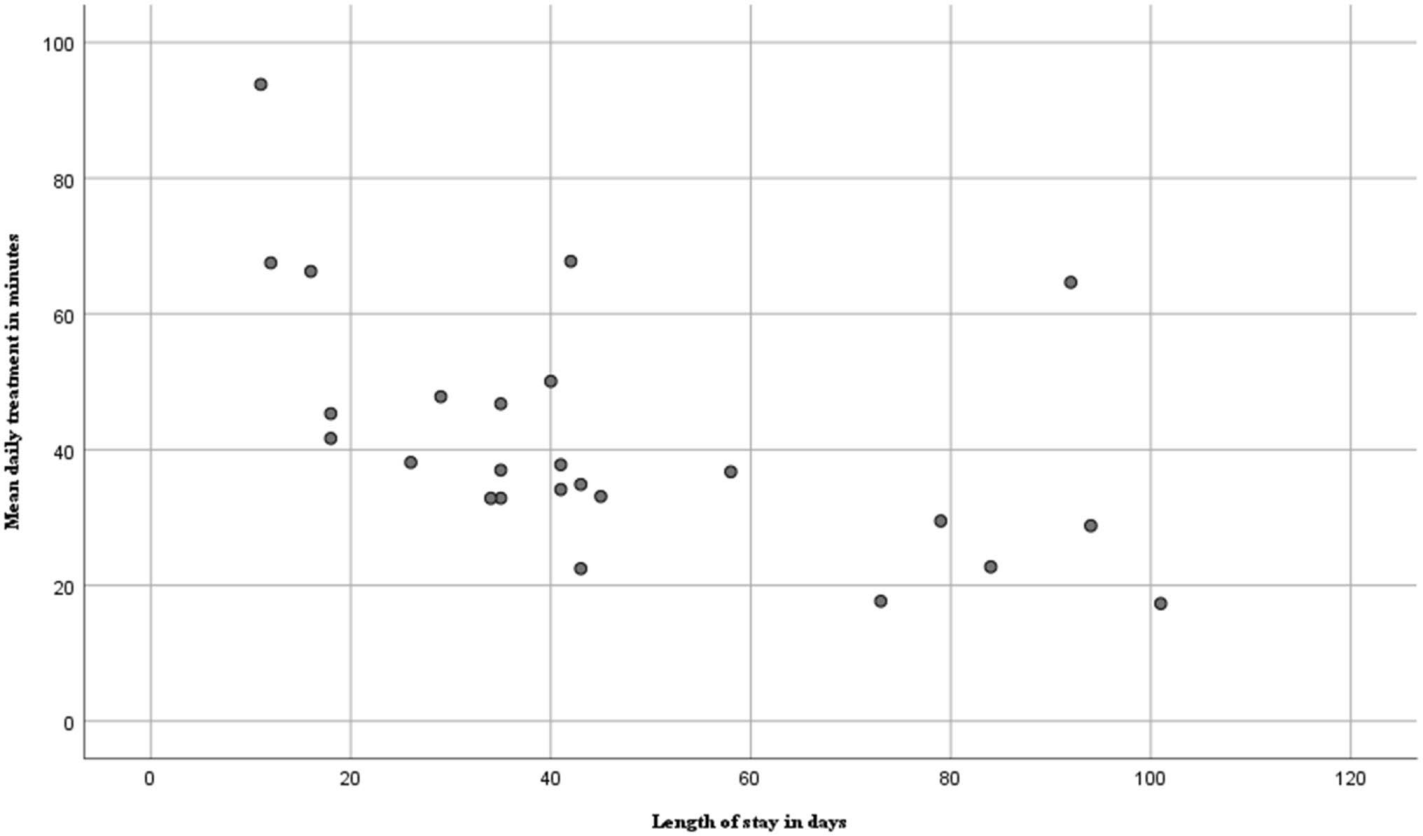
Mean daily treatment in minutes related to length of stay in geriatric rehabilitation facilities (*n* = 25 patients). Mean daily time is the total treatment time from all healthcare professionals per individual patient divided by their length of stay

Patients with a shorter length of stay received a higher amount of mean treatment time per day. The representation in Fig. 2 suggests that the treatment scheme of these patients was more intensive.

All 25 patients received care from the three key healthcare professionals in hip fracture rehabilitation (medical team, physiotherapist, and occupational therapist). According to the 25 DBC registrations, the dietician was consulted nine times, and the psychologist five times. No speech therapist or social worker was consulted for the 25 patients during their stay.

### Actual treatment time in DBC registration and allocated time in the care pathways

Only 25 DBC registrations were retrieved from seven GR facilities, one GR facility did not deliver their DBC registration. Of the 25 DBC registrations received, only 17 DBC registrations, contained registered therapy intensity per healthcare worker. A total of 12 DBC registrations, from four GR facilities, had corresponding care pathways, while one GR facility had five DBC registrations that registered average treatment time per week. Due to this registration variability, it was not possible to compare the care pathways with their own corresponding DBC registration. Table 2 shows, per healthcare professional per week, the range in allocated time according to care pathways and the mean treatment time according to the DBC registration for the 17 patients.

**Table 2:** Mean treatment time per geriatric rehabilitation facility (*n* = 8) in minutes per week as described in the care pathways compared to the actual mean time according to DBC registration of the hip fracture patients (*n* = 17)

	Allocated total treatment time per week in minutes (mean (SD)) according to the care pathways per geriatric facility									Actual treatment time per week in minutes (mean (SD)) according to DBC registration
	A (1–5 weeks)	B (1–6 weeks)	B (1–10 weeks)	C (1–6 weeks)	D (1–6 weeks)	E (1–6 weeks)	F (1–6 weeks) (1–10 weeks) (1–20 weeks)	G	H	DBC
Medical team										
First week	NA	180	170	120	120	120	NA	NA	NA	120 (59)
≥2 weeks	NA	36 (23)	27 (19)	34 (13)	30 (0)	15 (0)	NA	NA	NA	49 (29)
Physiotherapist										
First week	120	230	200	140	180	120	NA	NA	NA	157 (58)
≥2 weeks	120 (0)	179 (13)	119 (29)	74 (4)	140 (0)	90 (0)	NA	NA	NA	125 (50)
Occupational therapist										
First week	90	95	85	65	120	165	NA	NA	NA	93 (61)
≥2 weeks	30 (0)	40 (34)	33 (29)	25 (25)	60 (0)	60 (0)	NA	NA	NA	47 (44)

*SD* standard deviation, *NA* not available

For the medical team, the time allocated in the care pathways in the first week ranged from 120 to 180 min, while the DBC registration calculated a mean actual treatment time of 120 (SD 59) minutes. In weeks two to six, the allocated treatment time in the care pathways ranged from 15 to 36 min, while the DBC registration showed a mean actual treatment time of 49 (SD 29) minutes. For the physiotherapists, the allocated time in the care pathways ranged from 120 to 230 min while the mean actual treatment time in the first week was of 157 (SD 58) minutes. In weeks two to six, the allocated treatment time ranged from 74 to 179 min compared to the mean actual treatment time of 125 (SD 50) minutes. For the occupational therapist, the allocated time in care pathways ranged from 65 to 165 min in the first week, while the mean actual treatment time according to DBC registration was 93 (SD 61) minutes. In weeks two to six, allocated time ranged from 25 to 60 min compared to a calculated mean actual treatment time of 47 (SD 44) minutes.

## Discussion

This study describes the care provided for patients receiving rehabilitation after a hip fracture in GR facilities in the Netherlands. Six out of eight GR facilities had one or more written care pathways, which all differed in form and in content. All care pathways had three healthcare disciplines in common apart from the nursing staff, i.e., medical team, physiotherapy, and occupational therapy, but varied in time and/or frequency allocated per healthcare professional. Additional healthcare professionals such as dieticians, psychologists, and social workers were only mentioned in four GR facilities. Descriptions of assessment tools were available in four GR facilities, with the common assessment tool being the USER [23]. Screening for malnutrition and pain was only mentioned in three GR facilities, fall risks in two GR facilities, and polypharmacy in one GR facility. The DBC registrations confirmed a discrepancy between actual treatment time and allocated time as described in the care pathways.

### Care pathways and tools

As observed in our results, not all GR facilities implemented a care pathway. Care pathways, which are derived from or inspired by guidelines, are a method to standardize patient care management of a well-defined group of patients during a well-defined period of time [24]. Patients with a hip fracture are usually older persons with multimorbidity, who have complex problems from a biophysical and psychosocial perspective [25]. A care pathway may provide a structured process for rehabilitation in this vulnerable population. Care pathways contain information regarding the process of the care, such as involved professionals, the scheduling of multidisciplinary meetings, mandatory documentation, monitoring and evaluation of variance and outcomes. This structured process facilitates communication between team members and communication with patients and families [26]. Standardizing care pathways can also assist in improving quality of care, and, in addition, facilitate scientific research [27]. It should be noted that geriatric rehabilitation cannot always be standardized. Individual adaptation due to the diversity in patients must be taken in to consideration. Nevertheless, basic foundations such as assessment tools, and treatment principles need to be standard in care pathways, in order to measure the effectiveness of the rehabilitation [4]. Studies have been conducted to improve outcomes in patients with a hip fracture. The implementation of a multidisciplinary team has significant effect on shortening the length of stay in the hospital setting and on reducing mortality [4, 13, 28]. In our sample, only three health care professionals were consistent in the rehabilitation process. The dietician and psychologist were consulted in only four cases, showing minimal multidisciplinary involvement. By incorporating only the standard three healthcare professionals, the treatment scope tends to be limited to the somatic aspect, which is what we observed in the care pathways of the six GR facilities that used them. Literature recommends standardized screening for cognitive impairment, malnutrition [29, 30] and postoperative pain [31] to prevent stagnation during rehabilitation [29, 32–35]. Furthermore, by including a social worker in the multidisciplinary team to manage social as well as financial difficulties, earlier discharge can be achieved [34, 36].

Standardizing care pathways stimulates regular revisions based on ongoing research and updated guidelines. These revisions consist of fundamental recommendations to help healthcare professionals during the rehabilitation process. There is consensus on incorporating a multidisciplinary team specialized in the care of patients with hips fractures. A recent review analyzed interventions for improved functional outcome after hip fracture surgery. There was evidence that using an integrated multidisciplinary team, consisting of geriatricians, specialized nurses, occupational therapists, physiotherapists, psychologists, dieticians and social workers, yields better outcomes in terms of mortality and institutionalization [4]. International and national guidelines are based on evidence-based recommendations for the care of patients with a hip fracture. They emphasize intensity of physiotherapy, screening for cognitive impairment, fall risk prevention and efforts to minimize the risk of delirium to improve patient outcomes [9, 35, 37–43]. Moreover, several guidelines have also incorporated standardized detailed assessment tools for mobility and ADL [37, 44]. These assessments enable local care pathways to better assess the progression of the rehabilitation.

### Strengths and limitations

A strength of our study is that the patients originated from a hospital specializing in hip fractures. The patients were discharged to several GR facilities which had their own care pathways. The present study also has some limitations, such as a small sample of care pathways and DBC registrations collected in one region. However, in the Netherlands there is little difference throughout the regions [20]. Therefore, with this small sample size, we can give a good overview of the GR system in the Netherlands. Moreover, the DBC registrations offered no information about the quality of therapy, or external factors (such as comorbidity, pain, delirium, additional illness during rehabilitation or dementia) that may have affected total treatment time and length of stay. In addition, three patients were treated during the COVID pandemic which may have had an influence on the length of stay. In future research, it will be interesting to further investigate whether DBC registration depends on patient characteristics.

Nevertheless, to our knowledge, this is the first study in which care provided for patients with a hip fracture in GR facilities is explored based on available care pathways and actual registered treatment time per professional.

## Conclusion

Currently care provided in geriatric rehabilitation after hip fracture in the Netherlands is difficult to define due to the diversity in care pathways used. This is due to the fact that GR facilities use diverse care pathways and that care must be adapted for the individual needs and complications. Moreover, there is little consensus about time allocation, as well as on assessment instruments/screening needed to evaluate progression. More standardization of care pathways will support the implementation of the available evidence in GR facilities and will facilitate randomized effectiveness research. A standardized care pathway allows less variation in patient care and more clarity for healthcare professionals. Further research is needed to investigate whether a standardized care pathway is effective for GR.

## Data Availability

The datasets used and/or analyzed during current study are available from the corresponding author on reasonable request.

## Appendix A

See Table

**Table 3:** Treatment time per healthcare professional during rehabilitation after hip fracture in weeks, as described in care pathways (*n* = 9) from different geriatric rehabilitation facilities (*n* = 8)

Treatment time in minutes or frequencies per week								
GR facility Care pathway	Week 1^A^	Week 2	Week 3	Week 4	Week 5	Week 6	Week 7–10	Week 11–20
MT								
A	^a^	^a^	^a^	^a^	^a^			
B (1–6 weeks)^b^	180	20	20	20	70	50		
B (1–10 weeks)^b^	170	15	15	55	15	15	[15–55]	
C	120	55	35	20	30	30		
D	120	30	30	30	30	30		
E	120	15	15	15	15	15		
F (1–6 weeks)^b^	1×	1×	1×	1×	1×	1×		
F (1–10 weeks)^b^	3×	1×	1×	1×	1×	1×	1×	
F (1–20 weeks)^b^	3×	1×	1×	1×	1×	1×	1×	1×
G	NA	NA	NA	NA	NA	NA		
H	NA	NA	NA	NA	NA	NA		
PT								
A^c^	120	120	120	120	120			
B (1–6 weeks)^b^	230	170	170	170	185	200		
B (1–0 weeks)^b^	200	170	140	150	125	110	[90–95]	
C	140	70	75	70	75	80		
D	180	140	140	140	140	140		
E^c^	120	90	90	90	90	90		
F (1–6 weeks)^b^	4–6×	4–6×	4–6×	4–6×	4–6×	4–6×		
F (1–10 weeks)^b^	7×	2–5×	2–5×	2–5×	2–5×	2–5×	2–5×	
F (1–20 weeks)^b^	7×	2–5×	2–5×	2–5×	2–5×	2–5×	2–5×	2–5×
G	NA	NA	NA	NA	NA	NA		
H	NA	NA	NA	NA	NA	NA		
OT								
A	90	30	30	30	30			
B (1–6 weeks)^b^	95	80	15	15	75	15		
B (1–10 weeks)^b^	85	0	0	55	15	60	[0–65]	
C	65	60	10	0	10	45		
D	120	60	60	60	60	60		
E	165	60	60	60	60	60		
F (1–6 weeks)^b^	2–3×	2–3×	2–3×	2–3×	2–3×	2–3×		
F (1–10 weeks)^b^	4×	1–2×	1–2×	1–2×	1–2×	1–2×	1–2×	
F (1–20 weeks)^b^	4×	1×	1×	1×	1×	1×	1×	1×
G	NA	NA	NA	NA	NA	NA		
H	NA	NA	NA	NA	NA	NA		

*MT* medical team, *PT* physiotherapy, *OT* occupational therapy, *NA* not available

^a^No documented time/frequency in care pathway

^b^Same GR with different length of treatment

^c^In these houses, only the individual therapy was observed, not group therapy

^A^The first week of GR is associated with both admission assessments and initial treatment

3.

## Appendix B

See Table

**Table 4:** Assessment tools per GR facility (*n* = 8) as described in the care pathways

Assessment tools	GR facility							
	A	B^a^	C^a^	D	E	F	G^b^	H^b^
Utrecht Scale for Evaluation of Rehabilitation (USER)	×			×	×	×		
Canadian Occupational Performance Measure (COPM)	×			×	×			
Visual Analogue Scale (VAS)	×			×		×		
Short Nutritional Assessment Questionnaire (SNAQ)	×			×		×		
Time Up and Go (TUG)	×			×	×			
*Functional Ambulation Categories (FAC)*	×			×				
Patiënt Specifieke Klachten (PSK)^1^	×			×				
Berg Balance Scale (BBS)				×	×			
10 m walk test (10 MLT)	×			×				
Perceive Recall Plan Performed (PRPP)				×		×		
*Numeric (pain) rating scale (NPRS)*	×							
Elderly Mobility Scale (EMS)	×							
Tinetti	×							
Delirium Observation Scale (DOS)				×				
Allen Cognitive Level Screen (ACLS)				×				
Routine Outcome Monitoring (ROM)				×				
Dynamic Gate Index					×			
Assessment of Motor and Process Skills (AMPS)						×		
Activity Card Sort (ACS)						×		
Short Physical Performance Battery				×				
Fall analysis				×				
6 m walk test (6MWT)				×				
Falls Efficacy Scale (FES)	×							
Time Chair-Stand-Test (TCST)				×				
Hand Held Dynamometer (HHD)				×				
Borg Rating of Perceived Exertion Scale (BORG RPE)				×				
Mantelscan ^2^				×				
Ervaren Druk door Informele Zorg (EDIZ)^3^				×				
Self-Rated Burden scale (SRB)				×				
Caregiver Strain Index (CSI)					×			

^a^No assessment instruments in care pathways

^b^Care pathway not available

^1^No English translation. Measures difficulty in activity of the patients do to the complaints

^*2*^No English translation. Measure strengths and risk of the network

^3^No English translation. Measures stress experienced by the care giver

4.
